# A cryogenic probe for *in situ* delivery of gaseous samples for neutron scattering

**DOI:** 10.1107/S1600576726000543

**Published:** 2026-02-18

**Authors:** Chris Baldwin, Larissa Lopes Cavalcante, Nicholas D. Stapleton, Tait Francis, Tim D’Adam, Helen E. Maynard-Casely

**Affiliations:** a Australian Nuclear Science and Technology Organisation, Locked Bag 2001, Kirrawee DC 2232, Australia; b Department of Chemistry, University of Otago, Dunedin 9054, New Zealand; c School of Molecular Sciences, The University of Western Australia, 35 Stirling Highway, Crawley, Perth 6009, Western Australia, Australia; Oak Ridge National Laboratory, USA

**Keywords:** neutron scattering, gas delivery, diffraction, metal–organic frameworks, cryomineralogy

## Abstract

This laboratory note describes an optimized solution for the delivery of gaseous samples for neutron scattering measurements. The original design to accomplish this, though effective, was not ideal from a use and maintenance perspective – especially in view of the time-constrained nature of neutron scattering experiments. In response to user demand and a need for a more reliable solution the gas-delivery sticks have been significantly redesigned, with a number of innovations applied. Here we describe the resulting equipment and the operation of the gas sticks for condensation experiments, and we present results using this equipment and methodology.

## Abstract 2

We describe an optimized gas-delivery stick for use with top-loading cryostats for neutron scattering measurements.

## 1. Introduction

The ability to dose or condense gaseous species for study with neutron scattering is desirable across a range of applications, from porous materials to cryomineralogy. As a result, a system has been designed at the Australian Centre for Neutron Scattering to allow for delivering gases to samples *in situ* within a top-loading cryofurnace, which allows for temperature-dependent measurements between 4 and 800 K. The apparatus described in this contribution is a new design that has been optimized both for gas dosing to materials and now additionally for experiments on the condensed phases of gaseous species.

The first iteration of this system has been described previously (Lee *et al.*, 2016 ▸) and has led to a number of high-profile results on tuning thermal expansion with gas input (Auckett *et al.*, 2018 ▸), changing the thermal landscape (Chen *et al.*, 2023 ▸) and re-determining the crystal structure of nitrogen (Maynard-Casely *et al.*, 2020 ▸). However, the original design presented a number of challenges after entering user operations, proving difficult to maintain for continued operation.

The root of the challenges from the previous design was the detachable lower part of the stick, which can fit inside a glove box for the loading of air- and moisture-sensitive samples. This requires a valve (in this case a heavily modified Swagelok bellows valve with a heater to prevent blockages) and electrical connections for the heater loops on the valve, lower gas line and puck to be placed inside an insulating vacuum jacket – for a total of 18 connections. The accessible portion of the vacuum jacket was very small, so disconnecting and reconnecting the lower portion of the stick involved a complex and awkward process (often performed by visiting users), which frequently resulted in damage to the fine and delicate electrical connectors and subsequent loss of beam time to effect repairs. These challenges of use meant that in practice experimenters would often load their air-sensitive samples under solvent, before activating them by external heating of the sample can with the gas stick under vacuum. The detachable function of the previous design also presented challenges for condensing gases into the sample position, a growing demand at the instrument. This stemmed from the valve at the break between the stick sections, which was tight in the chamber preventing good thermal insulation. This issue was alleviated slightly by a low-wattage heater, but in practice the valve blocked very easily.

The lived experience with the previous design of gas stick was that experiments would normally succeed but with inevitable waste of neutron instrument time. This coupled with a large maintenance workload to keep customized parts in good use. Hence, a redesign was sought to reduce the reliance on customized parts, remove the detachable function and extend the use of the gas sticks for routine condensation experiments.

## 2. A new design of gas-delivery stick

The design philosophy of the second-generation gas-delivery probes was to prioritize reliability, repairability and modularity. The delivery-line and temperature-controlled puck assembly was designed such that it could be easily extracted from the outer vacuum-jacket assembly for repairs and maintenance. Multiple delivery-line assemblies were con­structed, allowing for a quick return to service in the event of damage. A benefit of this approach is that different delivery lines can be prepared in order to meet specific experimental needs. At the time of writing a large-inner-diameter line without provision for glove-box usage is adopted as standard, and a prototype glove-box loading variant (which will be the subject of a future report) has been planned.

**Figure 1:**
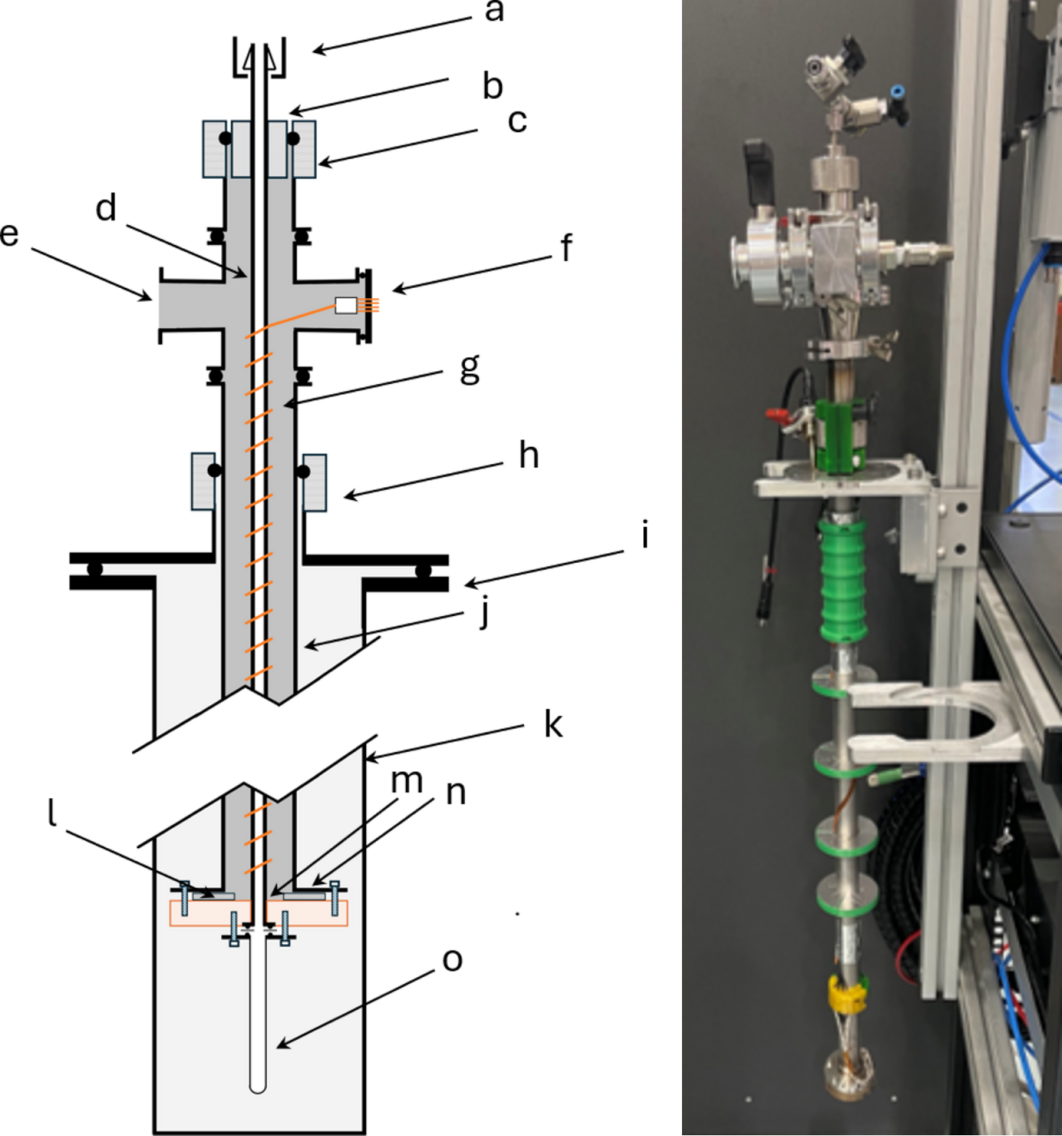
Schematic drawing of the new sticks (left). The letters correspond to (*a*) 1/4′′ swaged tube fitting, (*b*) 3/4′′ collar, (*c*) 3/4′′ o-ring compression vacuum fitting, (*d*) 1/4′′ delivery line, (*e*) KF16 vacuum port, (*f*) electrical vacuum feedthrough and vacuum side connector, (*g*) resistive heater wire, (*h*) flanged cryofurnace adapter, (*i*) cryofurnace flange, (*j*) vacuum jacket, (*k*) cryofurnace sample space, (*l*) PTFE gasket, (*m*) copper puck, (*n*) vacuum-jacket flange, and (*o*) vanadium sample can with welded VCR flange. Image of the stick without the vanadium sample can attached (right). The mountable cartridge heater for the bottom of the sample can is not shown in either image.

A schematic drawing is provided in Fig. 1 ▸.

The vacuum jacket [Fig. 1 ▸(*j*)] is constructed from a 25 mm seamless stainless steel tube: considerably larger and heavier than is typical for a cryogenic probe. A 3/4′′ collar [Fig. 1 ▸(*b*)] brazed to the delivery line enables the use of a large vacuum compression coupling (ProSciTech VCC-KF25-19) to couple to the vacuum jacket. This allows enough space for the 1/4′′ swaged fitting on the delivery line [Fig. 1 ▸(*a*)] to fit through the vacuum jacket for assembly and disassembly. Custom electrical connectors [Fig. 1 ▸(*f*)] were made (masked stereolithography 3D printed with commercial female pins, mating to a custom 12 pin M12 KF16 vacuum feedthrough) to facilitate easy disconnection.

The delivery line [Fig. 1 ▸(*d*)] is made from 6.35 mm stainless steel 316 seamless tubing with an inner diameter of 3.05 mm. The large inner diameter of the dosing line makes blockages due to condensation or deposition at cold spots much less common, and the tube is large enough to allow insertion of a 1.6 mm diameter tube from the top of the stick to within the sample can mounted below. This enables tube-in-tube gas-flow setups, as well as *in situ* liquid dosing with a syringe to the sample position. The pressure-bearing parts of the system are constructed from off-the-shelf components, with only one weld between the delivery line and knife-edge seal (Swagelok VCR) at the puck. The pressure rating of these components is in excess of 200 bar at room temperature. Allowable pressures are in practice determined by the pressure rating of the vanadium can, which in our case is at most 30 bar.

To provide heating to the delivery lines, Formvar insulated resistive heating wire is tightly wrapped around the the tube and secured with aluminium tape [Fig. 1 ▸(*g*)]. The heaters were separated into upper and lower zones, each with a PT100 resistance temperature detector for closed loop control. These are referred to subsequently as the upper gas line (UGL) and lower gas line (LGL) heaters.

Temperature control at the top of the vanadium sample can (sample-top temperature) is achieved with a temperature-controlled copper puck [Fig. 1 ▸(*m*)]. That at the bottom of the can is controlled by a sample space heat exchanger or an auxiliary heated puck that can be attached below the can. A 1/2′′ vacuum coupling radiation (VCR) gland (Swagelok SS-8-VCR-3-4MTW) is welded to the end of the delivery line, and brazed into the copper puck such that the knife edge is accessible from the bottom of the stick for attachment of the can [Fig. 1 ▸(*o*)]. The can has through holes in its lid and is attached to six M4 studs installed around the VCR fitting via nuts. Studs are used rather than screws to reduce the chance of stripping the soft copper threads in the puck. Helicoils have also been installed to reinforce the threads.

Screws attach the puck to the vacuum-jacket flange [Fig. 1 ▸(*n*)], with a polytetrafluoroethylene (PTFE) gasket [Fig. 1 ▸(*l*)] sandwiched between them to seal the vacuum jacket. To remove the delivery-line assembly, these screws are removed, the 3/4′′ compression fitting [Fig. 1 ▸(*c*)] is loosened and the electrical connector [Fig. 1 ▸(*f*)] is disconnected, and the puck and line assembly are pulled through the bottom of the vacuum jacket.

## 3. The gas sticks in use for condensing samples

The redesign was also a response to user demand for gas-condensing experiments, with the original sticks only being designed for gas dosing (for porous sample experiments *etc.*) (Lee *et al.*, 2016 ▸). The safety implication of having condensed gas that could (if the cryostat were to fail) boil rapidly meant that there was a need for overpressure valves to be included and, for co-condensing experiments, the ability to deliver liquid samples into the space too.

The basic principle of successful gas condensation in a cryostat is that conditions must only allow condensation within the sample can and not in any other part of the stick. If a liquid or solid blockage is formed in the delivery line, gas may be prevented from reaching the sample can. In the worst case, a solid blockage could cause an uncontrolled pressure build-up and rupture the system.

Gas-condensation experiments often involve hazardous compressed flammable gases. The authors advise anyone planning to re-create this apparatus to undertake a risk assessment before each experiment, specifically noting compatibility of gases with the components of the gas stick. A further key point is to not allow air into the gas-delivery manifold. This may result in blockages or even liquid/solid oxygen forming in the system. This can be alleviated by thorough leak checking and by keeping the system under vacuum prior to adding the sample gas – which is assisted by having a vacuum pump connected to the manifold.

### 3.1. Temperature profiles of the gas stick in use

The temperature profile of the delivery line during operation was measured by setting the delivery-line setpoint and then cooling the copper puck to various temperatures. The puck heater was used to maintain temperature – the cryofurnace heat exchanger was allowed to cool uncontrolled with heaters disabled. The temperature was allowed to equilibrate for at least two hours. A K-type thermocouple was then inserted into the delivery line to measure the internal temperature. Graduated markings were made on the insulating sheath every 50 mm to indicate the sensor’s position in the line. The thermocouple was inserted though a tee on the top of the delivery line with helium flowing from one port to exclude external moisture, and the line was capped between measurements.

**Figure 2:**
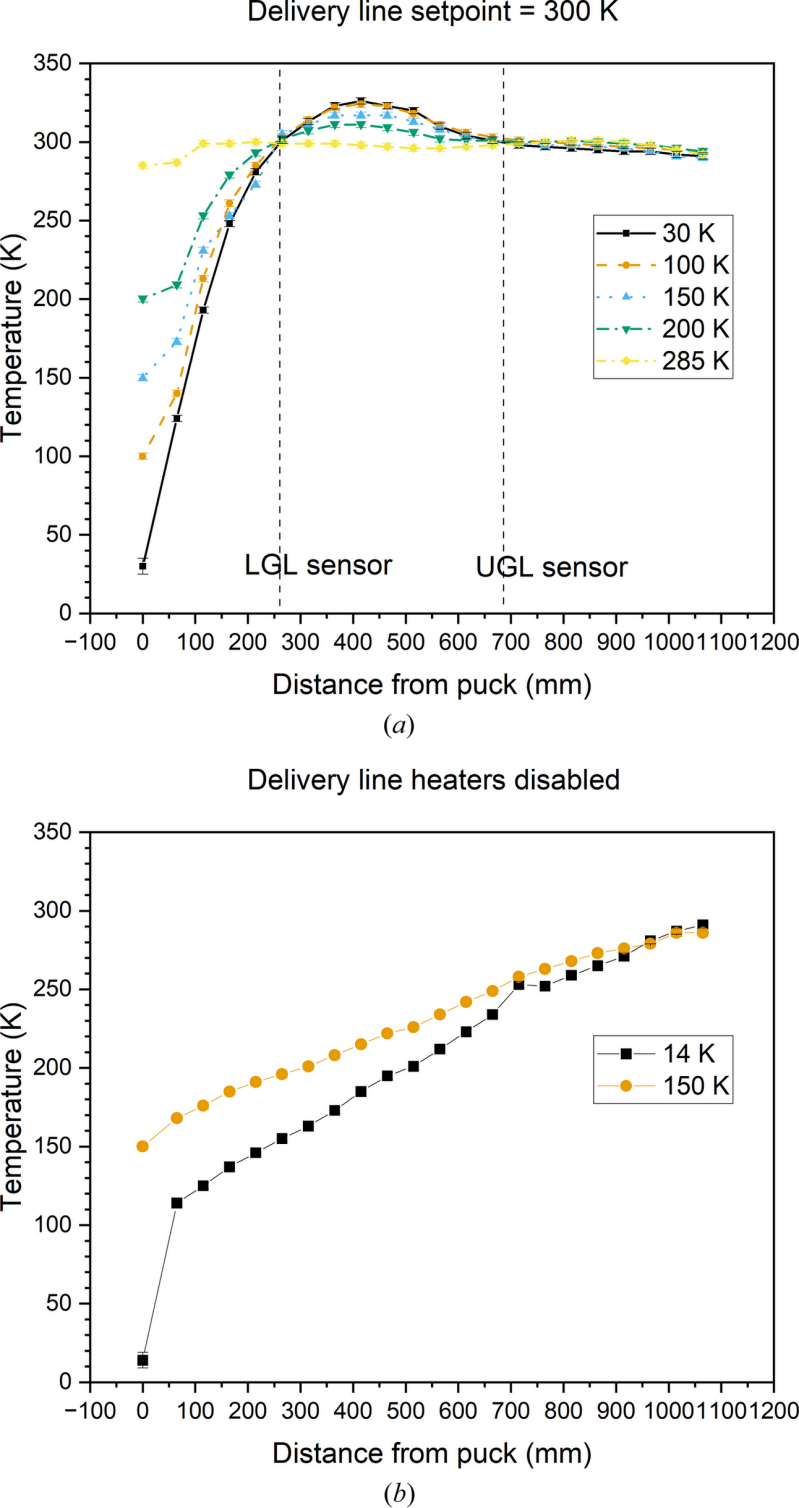
Delivery-line internal-temperature profiles with (*a*) UGL and LGL heaters set to 300 K and (*b*) UGL and LGL heaters disabled. Temperature labels indicate the sample-top temperature. For the 30 and 14 K profiles, the sample-top heater is disabled.

Fig. 2 ▸ shows the internal temperature profiles for the stick with delivery lines set to 300 K and disabled. With heaters enabled, the line is held at or above the setpoint for all points above the LGL sensor and transitions to the sample-top setpoint over the last 250 mm. With line heaters disabled, the temperature drops linearly over most of the length, with a sharp drop over the last few millimetres with the puck heater disabled. Notably, the sample top is the coldest point even with heaters disabled – there are no cold points along the line. Since condensation experiments require that the puck be warmer than the dew point (the condition where partial pressure is equal to vapour pressure), it is therefore possible to run a condensation experiment without delivery-line heaters. However, condensation can have significant safety consequences within the line, so the heaters are always used.

### 3.2. Methodology for condensing gaseous samples

The condensing setup requires that no cartridge heater is affixed to the lower part of the can, in order for thermal gradients to be established, thus enabling efficient condensation. As a result, temperature control is achieved through the heater affixed above the sample can [illustrated in Fig. 1 ▸, left, point (*l*) – known as the sample-top temperature] and the heat exchanger of the cryofurnace. The latter is thermally connected to the sample can with the inclusion of helium exchange gas.

Vapour-pressure curves should be consulted before attempting a condensation experiment. These can usually be estimated using the Antoine equation: 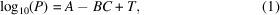
where *P* is the vapour pressure in bar and *T* is temperature in kelvin. *A*, *B* and *C* are empirical constants for a given gas and are available from the *NIST Chemistry WebBook* (Linstrom & Mallard, 2025 ▸) and elsewhere. Data for deuterated gases are unlikely to be available but can be estimated as the vapour pressure is generally a little lower than the hydrogenous counterpart, so use of hydrogenous curves can be effective along with conservative setpoints.

When the partial pressure of a gas exceeds its vapour pressure, condensation will occur. With a closed volume of gas, this will lower the partial pressure until it is equal to the vapour pressure, at which point condensation will stop. The difference Δ*P* between the initially applied pressure and the final pressure when condensation stops can be used to estimate how much liquid has formed in the can. Since the sample top, LGL and UGL must all remain above the dew point to prevent condensation in the delivery line, it is necessary that a thermal gradient is established between the sample top and the bottom of the can. A vanadium can in a cold 4 K cryofurnace with helium exchange gas will generally thermalize closely with the heat exchanger, almost independently of the sample-top heater. If one selects a heat-exchanger temperature with vapour pressure lower than the dosing pressure, then condensation is expected. The larger this gradient, the lower the final pressure when the dew point is reached and thus the larger Δ*P*.

**Figure 3:**
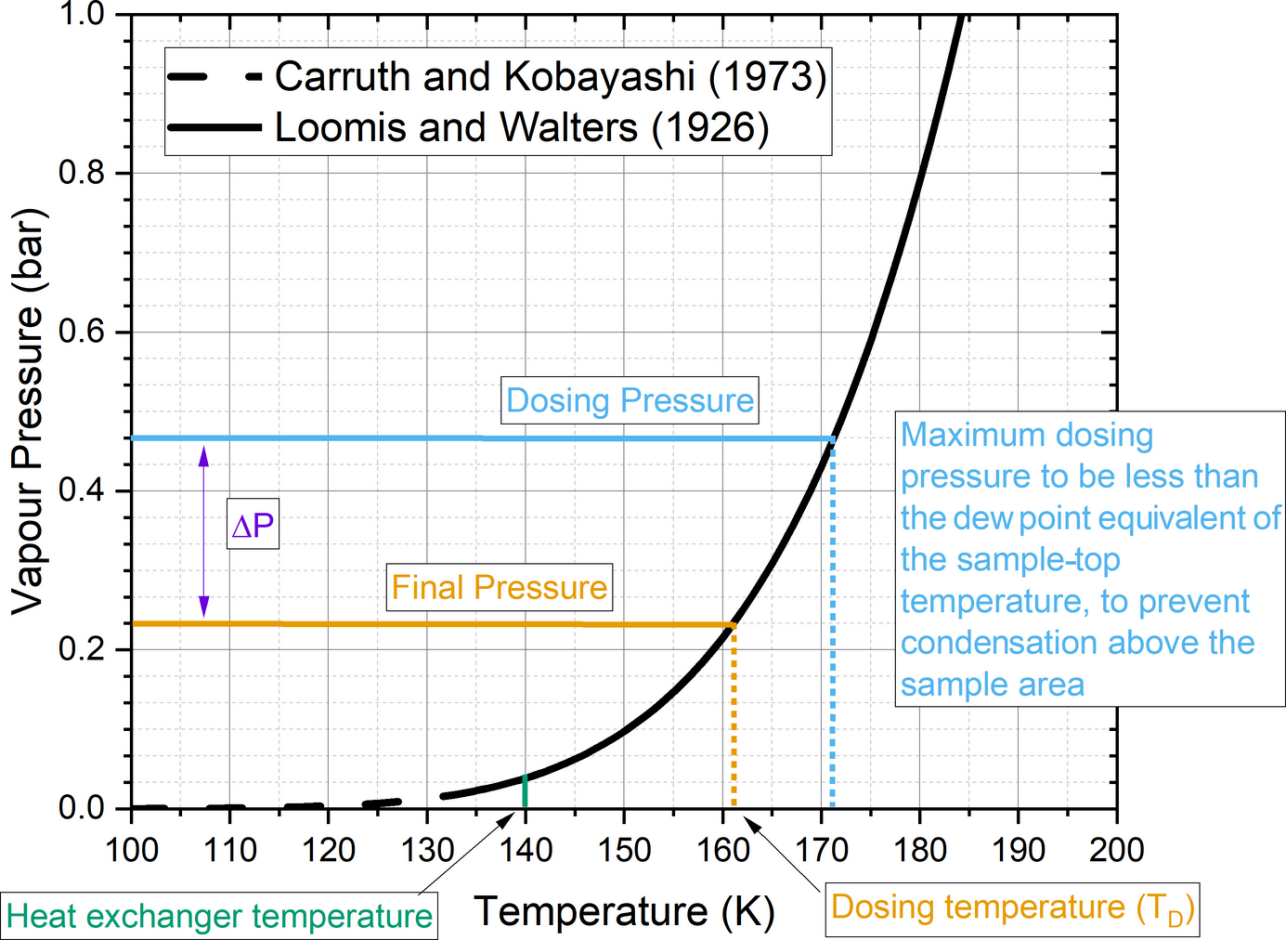
Example vapour-pressure curve for ethane with key quantities indicated. Heat-exchanger temperature and dosing pressure are selected for demonstrative purposes. The ethane vapour-pressure curve is plotted from Antoine equation coefficients from a NIST webbook derived from work by Carruth & Kobayashi (1973 ▸) and Loomis & Walters (1927 ▸).

However, once ‘warm’ gas is introduced during sample delivery, the heat load from cooling and condensation, as well as the thermal conduction path created to the sample top and delivery line by the gas, raises the temperature significantly higher than the heat-exchanger temperature. This may prevent any significant condensation from occurring. The final temperature of the coldest point of the can after a dose is referred to here as the dosing temperature (*T*_D_), which will be somewhere between the heat-exchanger temperature and sample top. *T*_D_ cannot be measured directly, but it can be inferred by tracing the final pressure in the gas-dosing volume into the vapour-pressure curve (Fig. 3 ▸).

Once sufficient sample has been condensed the stick volume is isolated from the manifold, and before the experiment begins the heat-exchanger and sample-top temperatures are set to minimize the gradient between them. Hence, during the experiment itself, *T*_D_ is not relevant. For subsequent experiments, variations of setting of heaters, temperature step, equilibration time and even gaseous environment of different experiments mean that it can be desirable to undertake temperature calibration using D_2_O for each unique setup and experiment profile, as was undertaken by Xiao *et al.* (2025 ▸).

## 4. Example of use – condensation of propene

**Figure 4:**
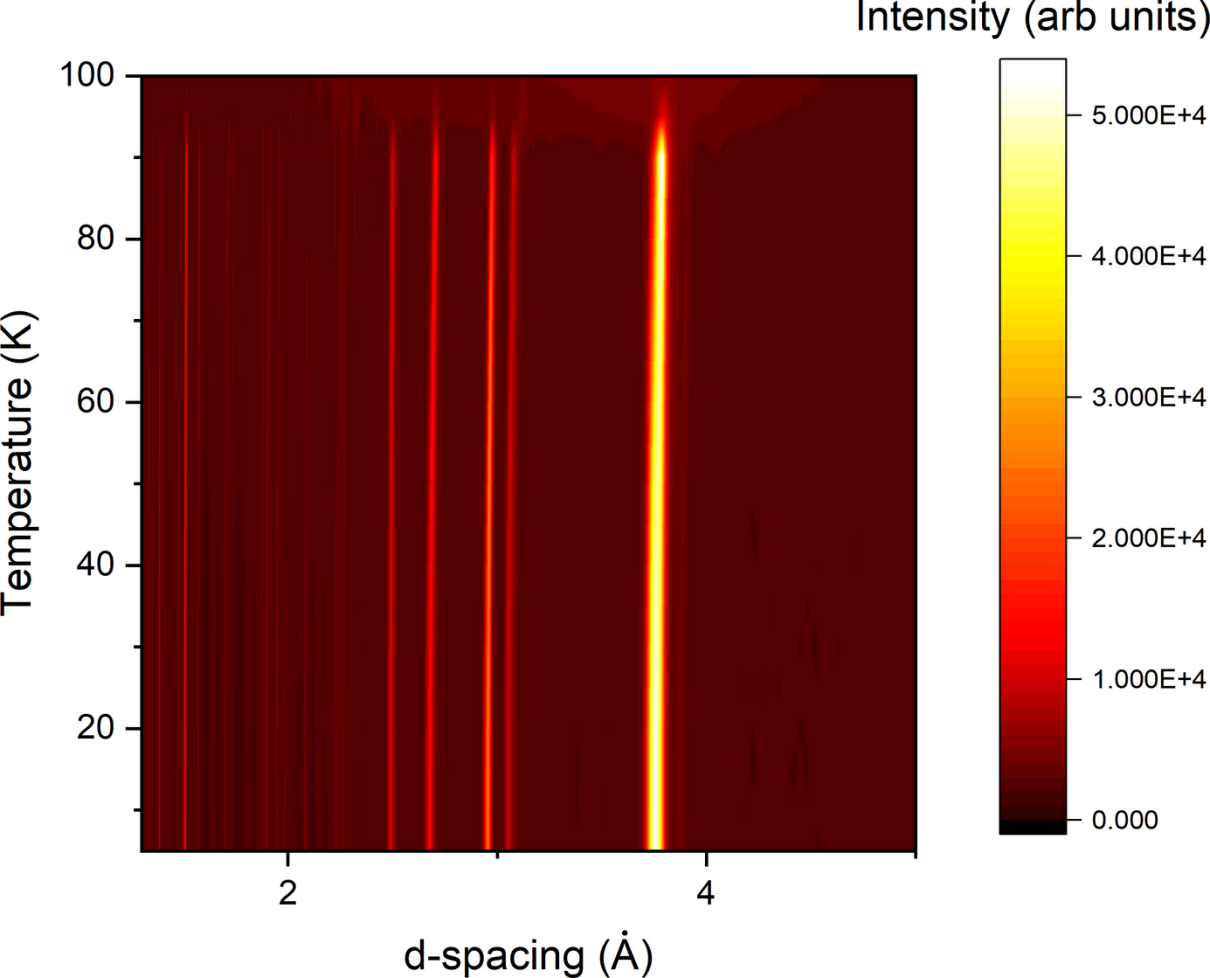
Thermodiffractogram of propene (CD_3_CD=CD_2_) studied with the gas-delivery sticks described in this contribution.

Propene (CH_3_CH=CH_2_, also known as propylene) is a raw product for a number of industrial uses, including the manufacturing of polypropylene and a common substitute for industrial MAPP gas (which is no longer in production). It is also one of the 18 simple organic compounds detected in the atmosphere of Saturn’s moon Titan (Yu *et al.*, 2023 ▸). However, to date there have been no diffraction studies of its solid phase, and there is no information in the literature on the crystalline structure it forms. Hence, the gas stick was used, with the procedure for condensing described above, to deliver a sample of d-propene (CD_3_CD=CD_2_, CDN Isotopes 99.8% D) into a 6 mm vanadium can for study on the Wombat high-intensity neutron diffractometer (Maynard-Casely *et al.*, 2026 ▸). The sample was cooled to base temperature (8 K, determined from the heat-exchanger temperature previously calibrated with D_2_O), and then a variable-temperature study was conducted between 8 and 100 K, with data collected at 2 K intervals; the resultant thermodiffractogram is plotted in Fig. 4 ▸.

Fig. 4 ▸ demonstrates that d-propene will crystallize to a single phase over the temperature range studied. The data were not of a sufficient resolution to allow for unit-cell indexing at this stage, but a follow-up experiment with higher-resolution instruments is planned. The collected data demonstrate the efficacy of the gas stick for undertaking novel experiments of condensed gaseous materials.

## 5. Conclusions

The redesigned gas sticks are now available as part of the user programme at the Australian Centre for Neutron Scattering. As the design is such that it can be used in a standard cryofurnace, the sticks can be used on a number of the instruments from the diffraction and inelastic suites. As well as the demonstrated example of d-propene, the gas sticks have proven effective in contributing to PhD theses of three of the authors, have allowed the delivery of argon for experimental support of equation of state determination (Xiao *et al.*, 2025 ▸) and have contributed to many more publications in preparation. From this reliable equipment base, future developments can be explored, including higher-pressure delivery, flow experiments and mixed-gas experiments.
